# Neurological Complications of COVID-19 and Possible Neuroinvasion Pathways: A Systematic Review

**DOI:** 10.3390/ijerph17186688

**Published:** 2020-09-14

**Authors:** Graziella Orrù, Ciro Conversano, Eleonora Malloggi, Francesca Francesconi, Rebecca Ciacchini, Angelo Gemignani

**Affiliations:** Department of Surgical, Medical and Molecular & Critical Area Pathology, University of Pisa, via Savi 10, 56126 Pisa, Italy; ciro.conversano@unipi.it (C.C.); e.malloggi1@studenti.unipi.it (E.M.); f.francesconi2@studenti.unipi.it (F.F.); rebecca.ciacchini@gmail.com (R.C.); angelo.gemignani@unipi.it (A.G.)

**Keywords:** COVID-19, SARS-CoV-2, neurologic complications

## Abstract

The Coronavirus Disease 2019 (COVID-19) outbreak has shocked the whole world with its unexpected rapid spread. The virus responsible for the disease, the Severe Acute Respiratory Syndrome Coronavirus 2 (SARS-CoV-2), enters host cells by means of the envelope spike protein, which binds to angiotensin-converting enzyme 2 receptors. These receptors are highly expressed in heart, lungs, respiratory tract epithelium, endothelial cells and brain. Since an increasing body of significant evidence is highlighting a possible neuroinvasion related to SARS-CoV-2, a state of the art on the neurological complications is needed. To identify suitable publications, our systematic review was carried out by searching relevant studies on PubMed and Scopus databases. We included studies investigating neurologic manifestations of SARS-CoV-2 in patients over 18. According to the analyzed studies, the most frequent disorders affecting central nervous system (CNS) seem to be the following: olfactory and taste disorders, ischemic/hemorrhagic stroke, meningoencephalitis and encephalopathy, including acute necrotizing encephalopathy, a rare type of encephalopathy. As regards the peripheral nervous system (PNS), Guillain-Barré and Miller Fisher syndromes are the most frequent manifestations reported in the literature. Important clinical information on the neurological manifestations of SARS-CoV-2 would help clinicians raise awareness and simultaneously improve the prognosis of critically ill patients.

## 1. Introduction

Since it was first announced in December 2019 in Wuhan, China, the Coronavirus Disease 2019 (COVID-19) has rapidly spread all over the world, and, on 11 March 2020, the World Health Organization (WHO) declared COVID-19 as a pandemic disease [1]. Genomic analyses showed that the virus responsible for COVID-19, the Severe Acute Respiratory Syndrome Coronavirus 2 (SARS-CoV-2), belongs to a beta-coronavirus cluster, along with the SARS-CoV and the Middle East Respiratory Syndrome Coronavirus (MERS-CoV) [2]. Infection in humans often leads to severe clinical symptoms and high mortality [3]. Clinical manifestations include respiratory symptoms and gastroenteric symptoms, with an incubation time ranging from two days to two weeks [4]. In particular, clinical characteristics of COVID-19 observed thus far are fever, dry cough, diarrhea, fatigue, dyspnea and pneumonia with peculiar lungs radiological features, such as bilateral multiple lobular and subsegmental areas of consolidations. Its severity ranges from asymptomatic manifestation to death [5]. Although much is known about the mortality rate and the main clinical manifestations of the disease, much less is known about its pathobiology. A probable course of events can be postulated according to past studies related to other coronavirus clusters (i.e., SARS-CoV). In this regard, evidence suggests that the virus enters host cells via the envelope spike (S) protein, which binds to angiotensin-converting enzyme 2 (ACE 2) receptor [6,7]. ACE 2 is known to be expressed in many organs, including heart, kidneys, liver, brain and abundantly in the epithelia of the lungs, respiratory tract, small intestine and endothelial cells [8]. Although SARS-CoV and SARS-CoV-2 have the same cellular target, SARS-CoV-2 shows higher binding affinity to ACE 2 [9]. There are other receptors that can mediate the entry of SARS-CoV-2 [7], e.g., transmembrane serine protease 2 (TMPRSS2) [10,11], sialic acid receptors [12,13] and extracellular matrix metalloproteinase inducer (CD147) [14]. Since COVID-19 was first identified in humans, scientific literature has dramatically increased, also reporting extra respiratory symptoms, among them neurologic complications. There is a huge body of evidence that points out to neurotropism as a characteristic of coronaviruses [6] and burgeoning studies have recently observed neurologic effects of SARS-CoV-2 on animal models, including non-human primates, mice, rats, hamsters and ferrets [15]. Furthermore, given the increasing amount of clinical data reporting neurologic involvement of SARS-CoV-2 [16,17] and post-mortem histologic studies revealing viral particles presence in human brain tissue [18], the urge to increase the awareness of the neurologic manifestations related to COVID-19 is of paramount importance. The purpose of the present study was to provide a systematic review of the literature, gathering relevant studies on the neurologic complications of COVID-19 on both the central nervous system (CNS) and the peripheral nervous system (PNS) in order to enhance a better management of infection-related neurological complications of affected patients.

## 2. Materials and Methods

**Figure 1:**
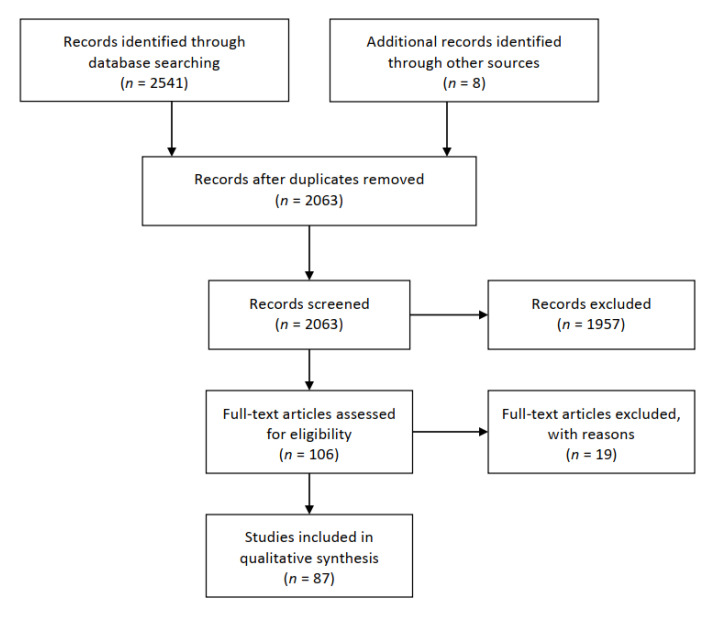
Flow chart of the studies selection.

This systematic review was carried out according to the Preferred Reporting Items for Systematic Review and Meta-Analysis guidelines (PRISMA, Figure 1) [19]. The search strategy consisted of three phases: (1) Identification: PubMed and Scopus databases were systematically searched in June 2020 with the following keywords: “brain” OR “nervous system” OR “central nervous system” OR “peripheral nervous system” OR “neurologic symptoms” OR “neurologic disorders” AND “COVID-19”. (2) Screening: A manual screening of the articles retrieved in phase 1 was conducted by evaluating titles and abstracts only. (3) Eligibility: Full-text articles were assessed on the basis of the following eligibility criteria: (i) individuals over 18 years old; (ii) case reports; (iii) retrospective studies; (iv) case-control studies; (v) in-press articles; and (vi) articles published in international peer-reviewed journals. In addition to the electronic databases search, references list of each study was scanned to ensure a comprehensive literature search. Only English-written articles were included. Uncorrected proofs were excluded.

## 3. Results and Discussion

Through the electronic databases search, 2541 publications were found. After manual removal of duplicates, 2063 articles were screened. Based on title and abstract reading, 1957 studies were excluded. In total, 106 full-text articles were assessed for eligibility. Among these, 19 studies were excluded for the following reasons: uncorrected proofs (*n* = 16); study protocol not started yet (*n* = 1); commentary study (*n* = 1); and vade mecum for neurologists (*n* = 1) to help them manage patients in the COVID-19 era. Finally, 87 studies met the inclusion criteria described above and were included (Figure 1).

### 3.1. Categorization and Characteristics of the Selected Studies

The studies included were subsequently divided into two categories: (1) studies concerning COVID-19 effects on the CNS (*n* = 68); and (2) those concerning COVID-19 effects on the PNS (*n* = 21), which are fully reported in the Supplementary Materials (Table S1 and Table S2). In total, 6890 patients were included in the studies. Among the 68 studies on the CNS effects, 46 were case reports [16,17,20,21,22,23,24,25,26,27,28,29,30,31,32,33,34,35,36,37,38,39,40,41,42,43,44,45,46,47,48,49,50,51,52,53,54,55,56,57,58,59,60,61,62,63], followed by 10 retrospective studies [64,65,66,67,68,69,70,71,72,73], 4 observational cohort studies [74,75,76,77], 3 prospective studies [78,79,80], 3 case-control studies [81,82,83] and 2 cross-sectional studies [84,85]. Among the 21 studies regarding PNS disorders, there were 19 case reports [86,87,88,89,90,91,92,93,94,95,96,97,98,99,100,101,102,103,104], 1 prospective study [78] and 1 retrospective study [68]. Two studies reported both CNS and PNS effects of COVID-19 [68,78].

Due to the vast heterogeneity of the studies design as regards to statistical validity, tools for assessing studies quality were used, based on “Algorithm for classifying study design for questions of effectiveness” (Algorithm for classifying study design for questions of effectiveness, (www.nice.org.uk): https://www.sign.ac.uk/assets/study_design.pdf).

#### 3.1.1. Effects of SARS-CoV-2 on CNS

Studies regarding CNS complications caused by SARS-CoV-2 infection are summarized in Table 1. Primary outcomes of the studies were classified in macrocategories regarding the main neurological complications.

An increasing body of significant evidence reported olfactory and taste disorders as specific clinical manifestations of SARS-CoV-2. In this context, a cross-sectional survey carried out by Giacomelli and colleagues [85] found that STDs were displayed in 20 out of 59 COVID-19 patients, most of whom were female and young. Twelve patients exhibited the symptoms at the onset of the disease and, among them, taste alterations were the most frequent (91%). These findings were mostly confirmed by Lechien and colleagues [66] in a European retrospective study: 85.6% and 88.0% of patients reported olfactory and gustatory dysfunctions, respectively, and a significant association between both disorders was detected (*p* < 0.001). The early olfactory recovery frequency was 44.0%. STDs prevalence was more common among females. It is worth noting that allergic rhinitis was the most frequent pre-existing medical condition among hyposmic and anosmic patients. These results were confirmed by a prospective cross-sectional study conducted by Speth and colleagues [80], in which the prevalence of olfactory disorders was around 61.2% and symptoms severity was correlated with the severity of taste disorders (*p* < 0.001). Furthermore, STDs strongly correlated with younger age (*p* = 0.007) and female sex (*p* = 0.56). Similarly, in their cross-sectional study, Dell’Era and colleagues [84] conducted a survey to detect chemosensory disorders prevalence among COVID-19 patients. They found that 249 out of 355 patients reported STDs; moreover, 31 of them reported these symptoms at the onset of COVID-19. Patients’ mean age was 50.

In their case-control study, Beltrán-Corbellini and colleagues [81] found STDs (especially anosmia) to be more present among COVID-19 patients (39.2%) compared to the control group (patients with influenza) (12.5%) (*p* = 0.003). Symptoms mean duration was one week with acute onset in 70.9% of the cases. COVID-19 patients with STDs were younger than those without STDs. Another case-control study carried out by Carignan and colleagues [82] described 69 patients with anosmia and 85 with dysgeusia out of 134 COVID-19 patients (mean age of 57.1). The frequency of chemosensory disorders was significantly higher among COVID-19 patients, compared to the control group (*p* < 0.001). Similarly, Hornuss and colleagues [83] detected a significantly higher prevalence of anosmia in a sample of COVID-19 patients (40% of 45 patients) as compared to control group (*p* < 0.001).

Spinato and colleagues [73] conducted a retrospective study reporting 130 patients out of 202 affected by STDs with different onset timing: symptoms occurred before other clinical manifestations in 11.9% of the patients, simultaneously with COVID-19 onset in 22.8% and after other symptoms in 26.7% of the subjects. STDs were the only symptoms of COVID-19 in 3.0% of the sample.

A prospective study, conducted by Lee and colleagues [79] on 3191 patients, reported STDs in 15.3% of patients at an early stage of COVID-19 and in 15.7% of asymptomatic patients or patients with mild disease severity. STDs were more frequently observed among females and young subjects.

Hopkins and colleagues [75] administered an online questionnaire to 382 COVID-19 patients to investigate the prevalence of smell and taste alterations and their course: 86.4% reported complete anosmia, which was almost reversed one week later in 80.1%, unchanged in 17.6% and worsened in 1.9% of the patients. Overall, 17.3% of the subjects reported persistent anosmia lasting from one to over four weeks.

Vaira and colleagues conducted two observational studies [76,77] to detect chemosensory dysfunctions among individuals who had contracted SARS-CoV-2 infection. In their first study, the authors recruited a sample of 72 COVID-19 patients treated at University Hospital of Sassari (mean age 49.2); among them, 53 reported STDs, 30 of whom reported both anosmia and dysgeusia. In the other study, the authors involved four Italian hospitals, reporting chemosensory disorders in 256 out of 345 patients. In total, 79.3% of them reported both olfactory and taste alterations, 8.6% isolated olfactory disorders and 12.1% isolated taste disorders.

These results are further supported by different case reports: seven studies [34,36,40,46,49,50] reported patients with olfactory and taste disorders as the only symptoms of COVID-19, apart from some subjects who also had other symptoms: myalgia, mild fever and cough [50], dyspnea, cough and chest tightness [46]. Most individuals were middle-aged and had no comorbidities, except for one subject who had atrial fibrillation and had undergone surgery for gastric ulcer [40] and another one who had hypertension, hyperlipidemia and asthma [46]. Patients mostly exhibited symptoms at the onset of the disease. Symptoms duration was 15 days on average.

The prevalence of the main neurologic features associated to COVID-19 was described by Karadaş and colleagues [78] in a cohort of 239 patients (mean age 46.46). Eighty-three patients displayed neurologic symptoms: headache was reported by 64 patients, dizziness in 16, 23 patients had impaired consciousness and 9 of them exhibited cerebrovascular disorders.

Lu and colleagues [67] carried out a multicenter retrospective study, enrolling 304 COVID-19 patients, 108 of whom had severe infection. Two patients had seizure-like symptoms during hospitalization, which were ascribed to acute stress reaction and hypocalcemia, and 84 had brain insults or metabolic imbalances.

In a cohort of 214 patients observed by Mao and colleagues [68], 78 had neurologic symptoms. Among them, symptoms were ascribable to CNS lesions such as dizziness, headache, impaired consciousness, acute cerebrovascular disease, ataxia and seizure (in order of prevalence). These patients were among those who displayed severe infection, older age, comorbidities, especially hypertension, and had fewer classical COVID-19 symptoms, compared to patients with mild disease severity.

Helms and colleagues [74] found neurological manifestations in 49 out of 58 patients admitted to hospital for acute respiratory distress syndrome caused by COVID-19. Patients exhibited agitation, confusion, corticospinal tract signs and dysexecutive syndrome. Magnetic Resonance Imaging (MRI) revealed leptomeningeal enhancement, perfusion abnormalities and cerebral ischemic stroke. Seven patients had pre-existing neurological conditions such as transient ischemic attack, partial epilepsy and mild cognitive impairment.

In their retrospective study, Kandemirli and colleagues [65] detected neurologic symptoms in 50 out of 235 COVID-19 patients admitted to intensive care unit, 27 of whom underwent MRI: 12 patients had abnormal MRI results; 10 of them had fluid-attenuated inversion recovery (FLAIR) signal abnormality and 2 had brain thrombosis and acute infarction.

MRI abnormalities were also reported by Radmanesh and colleagues [71] in 11 middle-aged patients with severe infection. Neurological complications included diffuse leukoencephalopathy and microhemorrhages in juxtacortical and callosal white matter. Patients had several pre-existing medical conditions, mostly regarding the cardiovascular system.

Two retrospective studies described critical stroke conditions related to COVID-19: Avula and colleagues [64] reported four patients exhibiting radiographic evidence of acute stroke, while Morassi and colleagues [69] observed five patients with ischemic and hemorrhagic stroke, with one patient developing encephalopathy before stroke; five of them died and one remained neurologically impaired. All subjects included in the two studies had previous vascular risk factors and their mean age was 81 in the former study and 69 in the latter.

Scullen and colleagues [72] carried out a retrospective study in which they categorized neurologic complications associated to COVID-19 based on brain imaging or electroencephalography (EEG) of 76 patients; 27 of them had positive neurological examination which revealed encephalopathy in 20 cases, acute necrotizing encephalopathy in 2 patients and vasculopathy in 5 patients.

An exhaustive systematic retrospective study was conducted by Petrescu and colleagues [70] who shed light on important EEG findings associated with SARS-CoV-2 infection. EEG was performed in 36 patients with an age ranging from 43 to 97 years. EEG recordings were normal to mildly altered in 57.5% of patients, while in 42.5% EEG alterations were observed: abnormalities were moderate in 10% of the patients, severe in 20% and critical in 12.5%. The most frequent comorbidities among patients were hypertension, diabetes mellitus, cardiomyopathy, renal failure and dementia.

The aforementioned findings are corroborated by a huge number of case reports.

Filatov and colleagues [30] reported a 74-year-old patient with encephalopathy and EEG abnormalities exhibiting inability to speak and follow commands as neurologic complications of COVID-19. The patient had several pre-existent cardiovascular pathologies and Parkinson’s disease. EEG abnormalities were detected by Flamand and colleagues, too [31]. The authors reported an 80-year-old woman exhibiting motor seizures; EEG revealed paroxysm with a triphasic aspect, suggesting a toxic/metabolic encephalopathy linked to COVID-19. Clonic seizures with loss of consciousness was reported as a COVID-19 onset symptom by Fasano and colleagues [29]. The patient was a 54-year-old man and had no relevant medical history. De novo status epilepticus was also reported by Somani and colleagues [56] in two COVID-19 patients. One patient was a 49-year-old woman with a past medical history of rheumatoid arthritis, schizoaffective and conversion disorder; the other patient was a 79-year-old woman who developed myoclonic status epilepticus with coma and then died. She had a past medical history of encephalocele and hydrocephalus. Brain imaging of both patients was unrevealing. Kadono and colleagues [41] also reported a case of seizures as secondary symptoms of SARS-CoV-2 infection. The patient was a 44-year-old man, who presented twitch on hand and face; furthermore, computerized tomography (CT) revealed right temporal lobe edema. The man had a past medical history of epilepsy following cerebral venous thrombosis. Similarly, Zanin and colleagues [61] reported the case of a 54-year-old woman exhibiting loss of consciousness and seizures concomitant with SARS-CoV-2 infection. Anosmia and ageusia were also present. MRI revealed demyelinating lesions, but no traces of virus RNA were found in CSF. The patient had a past medical history of anterior communicating artery aneurysm.

Moriguchi and colleagues [16] reported the first case of meningitis related to COVID-19 in a man of 24 years. Interestingly, SARS-CoV-2 RNA was not present in nasopharyngeal swab, but it was detected in cerebrospinal fluid (CSF) sample.

Several studies reported patients developing ischemic or hemorrhagic stroke secondary to SARS-CoV-2 infection. Viguier and colleagues [58] described a 73-year-old COVID-19 patient with no relevant medical history exhibiting acute ischemic stroke with aphasia and right hemiparesis as main clinical manifestations. Al-Saiegh and colleagues [20] described two patients affected by COVID-19 with concurrent neurologic symptoms. The first one, a 31-year-old man, exhibited headache and loss of consciousness, which were found to be clinical manifestations of subarachnoid hemorrhage. The other patient, a 62-year-old woman, had an ischemic stroke with hemorrhagic conversion. As already reported above with reference to another study [60], CSF samples were negative for SARS-CoV-2. Goldberg and colleagues [37] also reported the case of a 64-year-old man diagnosed with COVID-19 with concomitant acute ischemic stroke. He had a medical history of hypertension. Beyrouti and colleagues [24] reported six patients who exhibited multi-territory brain infarcts, venous thrombosis and ischemic stroke after a period ranging from 8 to 24 days from SARS-CoV-2 infection onset. Their mean age was 69.2 and most of them had cardiovascular risk factors. Tunç and colleagues [57] reported four patients aged between 45 and 77 affected by COVID-19 diagnosed with acute ischemic stroke. Three of them had a medical history of hypertension and the fourth one had diabetes. Deliwala and colleagues [27] described the case of a 31-year-old woman with no pre-existent clinical conditions exhibiting confusion and encephalopathy after diagnosis of COVID-19. Brain imaging revealed cortical ischemic stroke in right middle cerebral artery territory. Oxley and colleagues [51] reported five cases of large-vessel stroke in COVID-19 patients whose mean age was 40.4. Three of them (all males) had risk factors for stroke. Similarly, Barrios-López and colleagues [22] reported two male patients who developed ischemic stroke after SARS-CoV-2 infection. One patient was 50 years old and had a past medical history of diabetes mellitus and obesity. The other patient was 67 years old with no pre-existing pathologies. Other three studies described the cases of ischemic stroke concomitant with COVID-19: the patient reported by Co and colleagues [25] was a 62-year-old woman with several pre-existing pathologies such as hypertension, diabetes, dyslipidemia and transient ischemic attack; the patient reported by Frisullo and colleagues [33] was a young woman with no concomitant pathologies, while Zhai and colleagues’ [62] patient was a 79-year-old-man. Cerebrovascular complications caused by SARS-CoV-2 infection were also reported by Garaci and colleagues [35]. The authors described a 44-year-old woman with negative past medical history, who developed thrombosis of the superior vena cava, pulmonary artery and deep intracerebral venous thrombosis.

Numerous cases of encephalitis/encephalopathy associated with SARS-CoV-2 infection were reported, too. Ye and colleagues [60] observed a COVID-19 male patient exhibiting confusion and diagnosed with encephalitis; however, no traces of SARS-CoV-2 RNA in CSF were found. Duong and colleagues [28] reported the case of a 41-year-old woman diagnosed with meningoencephalitis as neurologic complication of COVID-19, showing disorientation and hallucinations. Symptoms evolved in lethargic status and then agitation. Reichard and colleagues [52] carried out post-mortem analysis in a 71-year-old man who died of COVID-19 complications, reporting acute disseminated encephalomyelitis and neocortical micro-infarcts. The patient had previously been diagnosed with ischemic heart disease caused by coronary artery atherosclerosis. Hayashi and colleagues [39] reported the first case of mild encephalitis/encephalopathy with a reversible splenial lesion in a 75-year-old-man with a history of mild Alzheimer’s disease. The first case of acute necrotizing encephalopathy as a complication of COVID-19 was reported by Poyiadji and colleagues [17]. The authors described a woman in her late fifties displaying altered mental status. Further investigation through brain imaging revealed hemorrhagic rim enhancing lesions within the bilateral thalami, medial temporal lobes and subinsular regions, coherent with the diagnosis of this rare type of encephalopathy. Furthermore, Zoghi and colleagues [63] described the case of a 21-year-old man who developed encephalomyelitis with atypical demyelination pattern revealed by MRI. The patient had negative medical history.

Kaya and colleagues [42] described a young man with COVID-19 presenting cortical blindness, apathia and inability to respond to commands. MRI and diffusion weighted imaging (DWI) revealed posterior reversible leukoencephalopathy (PRES). His medical history was negative for relevant pre-existing pathologies. Two cases of PRES concomitant with SARS-CoV-2 infection were also reported by Franceschi and colleagues [32]. Patients were a 48-year-old man with obesity and a 67-year-old woman with past medical history of hypertension, diabetes, coronary artery disease, gout and asthma. Another case of PRES was reported by Rogg and colleagues [53] in a 59-year-old COVID-19 patient who subsequently died. He had no relevant past history. Similarly, Kishfy and colleagues [43] described two COVID-19 patients (aged 58 and 67, respectively) who developed PRES. They had past medical histories of hyperlipidemia and of hypertension, diabetes and obesity.

A case of CSF negative for SARS-CoV-2, despite COVID-19 diagnosis with concomitant brain damage, was reported by Muhammad and colleagues [47]. The patient was a 60-year-old woman with left frontal subarachnoid hemorrhage exhibiting with loss of consciousness. Subarachnoid hemorrhage was also reported by Craen and colleagues [26] as a complication of COVID-19 in a 66-year-old woman with a past medical history of diabetes, hypertension and hyperlipidemia. A rare case of bilateral basal ganglia hemorrhage was detected by Haddadi and colleagues [38] in a 54-year-old woman who had contracted SARS-CoV-2 infection and with a past medical history of diabetes, hypertension, lumbar spinal laminectomy and fusion surgery. Microhemorrhages were revealed by MRI in corpus callosum, basal ganglia and brainstem in a 69-year-old COVID-19 patient with a history of hypertension, chronic kidney disease, and hypothyroidism reported by Shoskes and colleagues [55]. Sharifi-Razavi and colleagues [54] reported the case of a 79-year-old man exhibiting loss of consciousness caused by a massive intracerebral hemorrhage secondary to SARS-CoV-2 infection. He had no pre-existing medical conditions. Similarly, Benger and colleagues [23] reported a case series of five patients who developed intracerebral hemorrhage after contracting SARS-CoV-2 infection. They were aged between 41 and 64 and had several vascular risk factors, such as hypertension, past deep vein thrombosis and ischemic heart disease.

The case of a 36-year-old COVID-19-positive man presenting drowsiness and confusion was described by Al-olama and colleagues [21]. Symptoms were ascribable to meningoencephalitis complicated by intracerebral hematoma and subdural hematoma, revealed by brain imaging. CSF was found positive for SARS-CoV-2. No relevant medical history was reported.

Wong and colleagues [59] reported the case of a 40-year-old man who developed diplopia, limb ataxia, unsteady gait and nystagmus after contracting SARS-CoV-2 infection. MRI revealed brainstem inflammation, coherent with a diagnosis of rhombencephalitis. The patient had a medical history of glaucoma and hypertension. Brainstem lesion that led to depression of respiratory center was also reported by Manganelli and colleagues [44] in three patients. Two of them, a 66-year-old man and a 47-year-old woman, died a few days after the neurological examination.

Noro and colleagues [48] reported an interesting case of a fit 35-year-old woman who developed benign intracranial hypertension after contracting COVID-19. CSF was negative for SARS-CoV-2 infection.

Interestingly, Mawhinney and colleagues [45] reported the first case of acute manic episode associated with SARS-CoV-2 infection. In fact, the authors described a 41-year-old man who developed severe headache followed by sexual disinhibition, elevated mood and delusions ten days after COVID-19 symptoms onset. Neurological examination was negative. Twenty-three days later, the patient returned to baseline mental status. He had no comorbidities, but his sister had positive history for postpartum psychosis and was diagnosed with bipolar disorder.

**Figure 2:**
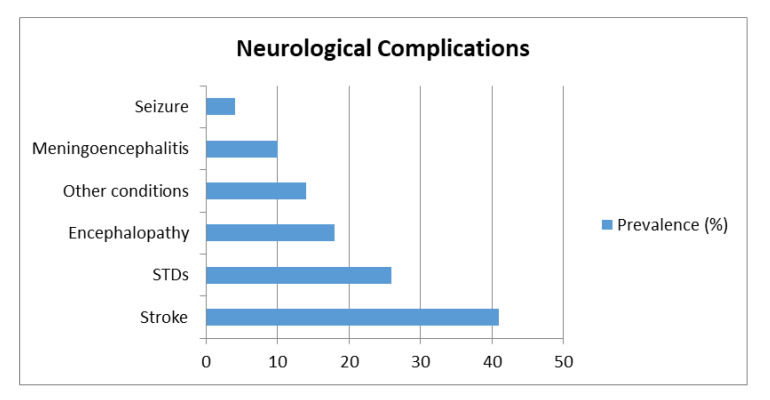
The prevalence of the main neurological complications of SARS-CoV-2 infection reported by the analyzed studies were: stroke (41%); STDs (26%); encephalopathy (18%); meningoencephalitis (10%); seizures (4%); and other conditions (14%), which included impaired consciousness, brain edema, headache, dizziness, acute mania, intracranial hypertension, EEG alterations, brain demyelination and vasculopathy.

Given the heterogeneity among the study designs, conducting a meta-analysis was not possible, but a preliminary analysis of the main neurologic complication prevalence reported by the studies was conducted and reported in Figure 2. The main neurologic conditions associated to SARS-CoV-2 were found to be stroke, STDs and encephalopathy. In particular, 41% of the studies reported stroke as neurological complication [20,22,23,24,25,26,27,33,35,37,38,44,47,51,52,54,55,57,58,62,64,65,67,68,69,71,74,78], 26% reported STDs [34,36,40,46,49,50,66,73,75,76,77,79,80,81,82,83,84,85] and 18% reported encephalopathy [17,30,31,32,39,42,43,53,67,71,72].

#### 3.1.2. Effects of SARS-CoV-2 on PNS

There is a less substantial but still relevant number of studies reporting neurologic complications of COVID-19 such as peripheral neuropathy, myopathy or peripheral demyelinating lesions.

In the aforementioned retrospective observational study carried out by Mao and colleagues [68], neurologic symptoms ascribable to PNS lesions were vision impairment, nerve pain and skeletal muscle pain.

The previously mentioned prospective study, conducted by Karadaş and colleagues [78], also reported peripheral nervous system complications among COVID-19 patients. The most frequent conditions were trigeminal neuralgia, glossopharyngeal neuralgia, vagoglossopharyngeal neuralgia, muscle pain, restless leg syndrome and Guillain–Barré syndrome.

Abdelnour and colleagues [86] reported the case of a 69-year-old man exhibiting numbness on both legs and reduced muscular power as secondary symptoms of SARS-CoV-2 infection. The patient had a past medical history of hypertension, diabetes mellitus and chronic obstructive pulmonary disease.

Homma and colleagues [92] reported the case of a 35-year-old patient who developed facial nerve palsy secondary to COVID-19. She had no pre-existing pathologies but she was a usual smoker.

Thirteen studies [87,88,90,93,95,96,97,98,99,100,101,102,104] reported an overall number of 17 patients aged between 21 and 71 with a diagnosis of Guillain–Barré syndrome as a complication of COVID-19. None of them had previous medical conditions, except for four patients: the one described by Alberti and colleagues [87] had hypertension, abdominal aortic aneurysm and lung cancer; the patient described by Hutchins and colleagues [93] had herpes simplex virus, hypertension, diabetes and obesity; the patient reported by Rana and colleagues [97] had hypertension, hyperlipidemia and restless leg syndrome; and the one reported by Webb and colleagues [102] had hypertension and psoriasis. When performed, CSF was found to be negative for SARS-CoV-2 infection [87,95,100,104].

Three studies [89,91,94] described Miller Fisher syndrome as another neurologic complication of SARS-CoV-2 infection. Dinkin and colleagues [89] reported two patients, a 71-year-old woman and a 36-year-old man, who developed also ophthalmoparesis. The former had pre-existing hypertension, while the latter had a past medical history of infantile strabismus. The two patients described by Gutierréz-Ortiz and colleagues [91] were two males aged 50 and 39, respectively, and had pre-existing bronchial asthma. Lantos and colleagues [94] reported a 36-year-old man with a medical history of left eye strabismus. All CSF swabs were negative for COVID-19.

Wei and colleagues [103] reported the case of a 61-year-old woman presenting oculomotor nerve palsy as a complication of SARS-CoV-2 infection. She had a past history of alcohol and tobacco addiction and pre-existing medical conditions such as hypertension, type II diabetes and lacunar infarction.

In conclusion, the aim of the present study was to investigate the neurological manifestations in COVID-19 patients, caused by SARS-CoV-2. In total, 86 studies were included and were divided into two categories: studies concerning COVID-19 effects on the CNS (*n* = 68) and those concerning the PNS (*n* = 21). In total, 6890 patients were included in our systematic review.

We observed a growing evidence of the neurological manifestations associated with COVID-19 involving the CNS and PNS.

According to the analyzed studies, the most frequent disorders affecting the CNS were the following: ischemic/hemorrhagic stroke [20,22,23,24,25,26,27,33,35,37,38,44,47,51,52,54,55,57,58,62,64,65,67,69,71,74], olfactory and taste disorders [34,36,40,46,49,50,66,73,75,76,77,79,80,82,83,84,85], meningoencephalitis [16,21,28,52,59,60,63] and encephalopathy [3,30,39,67,71,72].

As regards the PNS, the most frequent manifestations reported by literature were Guillain–Barré [78,87,88,90,93,95,96,97,98,99,100,101,102,104] and Miller Fisher syndromes [89,91,94].

SARS-CoV-2 neuro-invasiveness is a complex process that probably depends on multiple interacting factors such as viral load, tissue neurotropism and host immune response. Transgenic murine models for SARS-CoV provide a strong basis to help understand SARS-CoV-2 invasion into the nervous system: the virus enters the brain via the olfactory nerve and the cribriform plate of the ethmoid bone, on which the olfactory bulb lies. From this point, the virus spreads to connected brain areas [105]. Furthermore, ACE 2 and TMPRSS2 have been found to be expressed in sustentacular cells, stem cells and perivascular cells of the neuroepithelium, suggesting a crucial role of these cells in virus spread through the olfactory epithelium [106,107]. This mechanism may also explain why one of the most frequent symptoms of COVID-19 is anosmia, on which taste alteration depends [108].

Additionally, in CSF analysis, some studies reported traces of SARS-CoV-2 in the liquor [16,21], whereas others did not [20,60]. In this context, Al Saiegh and colleagues [20] proposed that, in the case of non-fulminant course of the disease, the brain–blood barrier may not be disrupted and consequently the virus does not infect CSF; furthermore, in the case of CSF negativity for SARS-CoV-2, neurologic symptoms might origin from an indirect pathogenic pathway which prevents CSF to be directly infected.

The studies analyzed thus far showed a significant heterogeneity and this aspect was particularly evident in: (1) the clinical profile of the population studied; (2) sample size; (3) study design; and (4) outcome measures employed.

Important clinical information on the neurological manifestations of SARS-CoV-2 would help clinicians raise awareness, and simultaneously improve prognosis of critically ill patients.

## 4. Conclusions

Due to the statistical heterogeneity among the analyzed studies, a meta-analysis investigation was not possible. Therefore, to minimize possible outcome biases, the adoption of case-control designs in future study protocols is strongly recommended.

Given the rapid evolution of the pandemic emergency and in the light of the increasing amount of clinical data reporting neurologic involvement in COVID-19, further investigations are required to better understand the underlying pathogenic mechanisms of SARS-CoV-2 neuro-invasion. Furthermore, thorough knowledge of the main neurologic features associated with COVID-19 is of particular importance, since some hypotheses have been proposed regarding a possible primary role of the nervous system in determining respiratory symptomatology. Finally, we strongly suggest developing systematic psychological, neurological and neuropsychological examinations of affected patients and efficient follow-up treatments, in order to manage possible long-term effects of neurologic complications, especially in the case of patients with pre-existing cerebrovascular risk factors, and to alleviate the burden of critically ill patients. The Inter-Agency Standing Committee guidelines for mental health and psychosocial support during emergency situations recommend integration of multiple levels of intervention in response to epidemics [109,110,111,112,113].

This could prevent neurodegenerative processes induced by neuroinflammation or stroke-dependent permanent neurologic deficits. Further elucidations in this regard are needed, in order to better understand COVID-19 etiopathogenesis and to better handle COVID-19 patients.

## Figures and Tables

**Table 1:** Categorization of the studies concerning SARS-CoV-2 effects on the CNS: (a) Stroke; (b) Smell and taste disorders (STDs); (c) Encephalopathy; (d) Meningoencephalitis; (e) Seizures; and (f) Other Neurological Conditions.

Article/Sample (*n*)	*p*-Value/rho Value/Odd Ratio
**(a) Stroke**	
Al Saiegh et al. [20] *n* = 2	N.A.
Avula et al. [64] *n* = 4	N.A.
Barrios-Lòpez et al. [22] *n* = 4	N.A.
Beyrouti et al. [24] *n* = 6	N.A.
Benger et al. [23] *n* = 5	N.A.
Co et al. [25] *n* = 1	N.A.
Craen et al. [26] *n* = 1	N.A.
Deliwala et al. [27] *n* = 1	N.A.
Frisullo et al. [33] *n* = 1	N.A.
Garaci et al. [35] *n* = 1	N.A.
Goldberg et al. [37] *n* = 1	N.A.
Haddadi et al. [38] *n* = 1	N.A.
Helms et al. [82] *n* = 58	N.A.
Kandemirli et al. [65] *n* = 23	N.A.
Karadaş et al. [74] *n* = 239	N.A.
Lu et al. [67] *n* = 304	N.A.
Manganelli et al. [44] *n* = 3	N.A.
Mao et al. [68] *n* = 214	Stroke prevalence (*p* = 0.03); Major Complications (*p* = 0.02)
Morassi et al. [69] *n* = 6	N.A.
Muhammad et al. [47] *n* = 1	N.A.
Oxley et al. [51] *n* = 5	N.A.
Radmanesh et al. [71] *n* = 11	N.A.
Reichard et al. [52] *n* = 1	N.A.
Sharifi-Razavi et al. [54] *n* = 1	N.A.
Shoskes et al. [55] *n* = 1	N.A.
Tunç et al. [57] *n* = 4	N.A.
Viguier et al. [58] *n* = 1	N.A.
Zhai et al. [62] *n* = 1	N.A.
**(b) STDs**	
Beltrán-Corbellini et al. [81] *n* = 79	*p* = 0.003: STDs more frequent among cases
Carignan et al. [82] *n* = 134	*p* < 0.001: STDs more frequent among cases as compared to controls
Dell’Era et al. [84] *n* = 355	*p* < 0.001: full recovery after 14 days
Gane et al. [34] *n* = 1	N.A.
Giacomelli et al. [85] *n* = 59	*p* < 0.001: association between anosmia and dysgeusia
	*p* = 0.036: STDs more frequent among F
	*p* = 0.035 STDs more frequent among younger patients
Gilani et al. [36] *n* = 8	N.A.
Hjelmeseth and Skaare [40] *n* = 60	N.A.
Hopkins et al. [75] *n* = 382	*p* < 0.001: symptoms improvement after 2 weeks
Hornuss et al. [83] *n* = 45	*p* < 0.001: STDs more frequent among cases
Lechien et al. [66] *n* = 417	*p* < 0.001: anosmia and dysgeusia and more frequent among F
Lee et al. [79] *n* = 3191	*p* = 0.01: more common in F
	*p* < 0.001 more common in young patients
Melley et al. [46] *n* = 1	N.A.
Ollarves-Carrero et al. [49] *n* = 1	N.A.
Ottaviano et al. [50] *n* = 6	N.A.
Speth et al. [80] *n* = 103	*p* < 0.001: prevalence of STDs
	*OR* = 0.96: negative correlation between STDs and older age
	*OR* = 2.46: positive correlation between STDs and F
	*ρ* = 0.87: correlation between severity of taste and olfactory disorders
Spinato et al. [73] *n* = 202	*p* = 0.02: STDs more frequent among women
Vaira et al. [76] *n* = 72	*p* = 0.003: taste disorders worse in older patients (age ≥ 50)
	*p* = 0.001: STDs improvement after 15 days from the onset
Vaira et al. [77] *n* = 345	*p* = 0.000: symptoms improvement after one or two weeks
**(c) Encephalopathy**	
Filatov et al. [30] *n* = 1	N.A.
Flamand et al. [31] *n* = 1	N.A.
Franceschi et al. [32] *n* = 2	N.A.
Hayashi et al. [39] *n* = 1	N.A.
Kaya et al. [42] *n* = 1	N.A.
Kishfy et al. [43] *n* = 2	N.A.
Lu et al. [67] *n* = 304	N.A.
Poyiadji et al. [17] *n* = 1	N.A.
Radmanesh et al. [71] *n* = 11	N.A.
Rogg et al. [53] *n* = 1	N.A.
Scullen et al. [72] *n* = 76	N.A.
**(d) Meningoencephalitis**	
Al-olama et al. [21] *n* = 1	N.A.
Duong et al. [28] *n* = 1	N.A.
Moriguchi et al. [16] *n* = 1	N.A.
Reichard et al. [52] *n* = 1	N.A.
Wong et al. [59] *n* = 1	N.A.
Ye et al. [60] *n* = 1	N.A.
Zoghi et al. [63] *n* = 1	N.A.
**(e) Seizures**	
Fasano et al. [29] *n* = 1	N.A.
Lu et al. [67] *n* = 304	N.A.
Somani et al. [56] *n* = 2	N.A.
(f) Other Neurological Conditions *	
Kadono et al. [41] *n* = 1	N.A.
Karadas et al. [78] *n* = 239	N.A.
Mawhinney et al. [45] *n* = 1	N.A.
Noro et al. [48] *n* = 1	N.A.
Petrescu et al. [70] *n* = 36	N.A.
Scullen et al. [72] *n* = 76	N.A.
Zanin et al. [61] *n* = 1	N.A.
Mao et al. [68] *n* = 214	*p* < 0.001

Notes: N.A., not applicable; *p*, *p*-value; *ρ*, rho value. F, female; *OR*, odd ratio. * Other Neurological Conditions were the following: brain edema [41]; headache [78]; impaired consciousness [68,78]; dizziness [78]; acute mania [45]; intracranial hypertension [48]; electroencephalography alterations [70]; vasculopathy [72]; and brain and spine demyelination [61].

## Supplementary Materials

The following are available online at https://www.mdpi.com/1660-4601/17/18/6688/s1, Table S1: Studies concerning COVID-19 effects on the Central Nervous System; Table S2: Studies concerning COVID-19 effects on the Peripheral Nervous System.
