# Supplementary material for: Neurological Complications of COVID-19 and Possible Neuroinvasion Pathways: A Systematic Review
Source: Int J Environ Res Public Health. 2020 Sep 14;17(18):6688. doi: 10.3390/ijerph17186688 (PMC7558767; doi:10.3390/ijerph17186688)
Supplement: Supplementary file 1 [file ijerph-17-06688-s001.pdf]

Table S1. Studies concerning COVID-19 effects on the Central Nervous System.

Article	Study design	Sample	Age	Country	Neurological Complications	Prior Medical Conditions
Al-Saiegh et al., (2020) [20]	Case report	N=2<br>M=1<br>F=1	M=31<br>F=62	USA	-Patient 1: H&H grade 3 aneurysmal SAH<br>-Patient 2: ischemic stroke with massive hemorrhagic conversion<br>-Both patients' conditions evolved into hydrocephalus, but CSF was negative for SARS-CoV-2	None
Al-olama et al., (2020) [21]	Case report	N=1<br>M	36	United Arab Emirates	-Meningoencephalitis complicated by intracerebral hematoma and subdural hematoma<br>-CSF proved positive for SARS-CoV-2	None
Avula et al., (2020) [64]	Retrospective study	N=4<br>M=1<br>F=3	81	USA	Acute stroke	Patient 1: hypertension, dyslipidemia, carotid stenosis<br>Patient 2: frequent urinary tract infections, hypertension, hyperlipidemia, diabetes mellitus type 2, neuropathy<br>Patient 3: hypertension<br>Patient 4: hypertension, chronic kidney disease, hyperlipidemia

Barrios-Lòpez et al., (2020) [22]	Case report	N=4<br>M=2<br>F=2	71.5	Spain	-Patient 1: stroke in the distal territory of the left MCA<br>-Patient 2: stroke in the distal portion of the left superior cerebellar artery and left posterior cerebral artery<br>-Patient 3: subacute ischemic stroke in the right hemisphere<br>-Patient 4: Right hemisphere stroke with occlusion of the MCA	Patient 1: diabetes mellitus type 2, former smoker, obesity<br>Patient 2: None<br>Patient 3: hypertension, diabetes mellitus type 2, hypertensive heart disease, asthma<br>Patient 4: hypertension, diabetes mellitus type 2, permanent atrial fibrillation, ischemic heart disease
Beyrouti et al., (2020) [24]	Case report	N=6<br>F=1<br>M=5	69.2	UK	-Patient 1: extensive acute posterior cerebral artery territory infarction with consequent bilateral incoordination and right homonymous hemianopia<br>-Patient 2: acute large left cerebellar and right parieto-occipital infarcts with consequent confusion, incoordination and drowsiness<br>-Patient 3: left posterior cerebral artery occlusion and infarction with consequent dysarthria and right hemiparesis<br>-Patient 4: acute right striatal infarct manifesting with dysarthria, left facial droop and left-sided weakness	Patient 1 and 6: none<br>Patient 2: Hypertension, diabetes, mitral valve replacement, atrial fibrillation, heart failure with a permanent pacemaker<br>Patient 3: Hypertension, hypercholesterolemia, atrial fibrillation, ischemic heart disease, prostate cancer

					-Patient 5: thrombotic occlusion of a branch of the right middle cerebral artery with dysarthria and left hemiparesis<br>-Patient 6: thrombus in the basilar artery, bilateral P2 segment stenosis and multiple acute infarcts with consequent dysphasia and right hemiparesis	Patient 4: hypertension, previous stroke and high body mass index<br>Patient 5: hypertension, diabetes, ischemic heart disease, heavy smoking and alcohol consumption
Beltrán-Corbellini et al., (2020) [81]	Case-control study	N= 79<br>M=48<br>F=31<br><br>CG= 40<br>M=19<br>F= 21	61.6±17.4<br><br><br><br>61.1±17.1	Spain	-31 STDs among cases and 8 among controls were found<br>-COVID-19 patients with new-onset STDs were younger than COVID-19 patients without STDs	None
Benger et al., (2020) [23]	Case report	N=5<br>M=3<br>F=2	52.2	UK	-Patient 1: left frontal intracerebral hemorrhage with drowsy and right-sided hemiplegia<br>-Patient 2: right frontal lobe intracerebral hemorrhage with dysarthria and left sided hemiparesis<br>-Patient 3: right frontal lobe intracerebral hemorrhage<br>-Patient 4: subacute right-sided gangliocapsular intracerebral hemorrhage<br>-Patient 5: subacute intracerebral hemorrhage in the right perirolandic region with smaller petechial haemorrhages in the left paramedian frontal lobe and left cingulate sulcus	Patient 1: hypertension and type 2 diabetes mellitus Patient 2: multiple deep vein thrombosis, pulmonary embolism, hypertension and type 2 diabetes mellitus Patient 3: hypertension Patient 4: none Patient 5: ischemic heart disease, quiescent untreated multiple sclerosis, asthma, hypertension and hyperlipidemia

Carignan et al., (2020) [82]	Age-matched case-control study	N=134 M=64 F=70 CG=134	57.1 (42.6–64.4)	Canada	-69 patients exhibited anosmia and 85 patients showed dysgeusia<br>-frequency of STDs was higher among COVID-19 patients as compared to CG	N.A.
Co et al., (2020) [25]	Case report	N=1 F	62	Philippines	Ischemic stroke with hypodensity in the left semioval centre and corona radiata and stenosis of the MCA, presenting with dysarthria and upper and lower extremity weakness	Hypertension, diabetes, dyslipidemia, transient ischemic attack
Craen et al., (2020) [26]	Case report	N=1 F	66	Usa	Subarachnoid haemorrhage manifesting with altered mental status	Diabetes, hypertension and hyperlipidemia
Deliwala et al., (2020) [27]	Case report	N=1 F	31	USA	Cortical stroke in the region of MCA	None
Dell'Era et al., (2020) [84]	Cross-sectional study	N=355 M=192 F=163	50.5 (40-59.5)	Italy	-249 patients reported STDs<br>-31 patients showed symptoms at the onset of COVID-19	Cardiac comorbidities (N=42)<br>Respiratory comorbidities (N=40)<br>Allergic/nasosinusal comorbidities (N=38)
Duong et al., (2020) [28]	Case report	N=1 F	41	USA	Meningoencephalitis manifesting with hallucinations and disorientation	Obesity, diabetes
Fasano et al., (2020) [29]	Case report	N=1 M	54	Italy	Focal motor seizure	None
Filatov et al., (2020) [30]	Case report	N=1 M	74	USA	Encephalopathy and epileptiform discharges in the right temporal region manifesting with inability to speak and follow commands	Atrial fibrillation, cardioembolic stroke,

						Parkinson's disease, COPD and recent cellulitis
Flamand et al., (2020) [31]	Case report	N=1 F	80	France	Triphasic waves in EEG	N.A.
Franceschi et al., (2020) [32]	Case report	N=2 M=1 F=1	48 M 67 F	USA	PRES	Patient 1: obesity<br>Patient 2: hypertension, diabetes, coronary artery disease, gout and asthma
Frisullo et al., (2020) [33]	Case report	N=1 F	49	Italy	Two small acute brain infarctions in the right perirolandic cortex	N.A.
Gane et al., (2020) [34]	Case report	N=1 M	48	UK	Anosmia	None
Garaci et al., (2020) [35]	Case report	N=1 F	44	Italy	Thrombosis of the superior vena cava, pulmonary artery and deep intracerebral venous thrombosis	None
Giacomelli et al., (2020) [85]	Cross-sectional study	N=59 M=40 F= 18	60 (50-74)	Italy	20 patients with STDs were found:<br>-91% of dysgeusia symptoms occurred before hospitalization<br>-31 were females<br>-12 patients showed STDs as onset symptoms<br>-8 patients exhibited symptoms after hospitalization<br>-Mean age was 56<br>-An association between disorders was found	N.A.

					-Females were more affected by both smell and taste disorders	
Gilani et al., (2020) [36]	Case report	N=8<br>M=2<br>F=6	35.4	Iran	Onset olfactory and taste loss	Rhinoplasty (N=3)
Goldberg et al., (2020) [37]	Case report	N=1<br>M	64	USA	Cerebral edema and infarcts of the right MCA and bilateral ACA territories manifesting with left-sided hemiparesis	Hypertension, aplastic anemia, splenectomy.
Haddadi et al., (2020) [38]	Case report	N=1<br>F	54	Iran	Bilateral subacute hemorrhage in basal ganglia and altered mental status	Diabetes, hypertension, lumbar spinal laminectomy and fusion surgery
Hayashi et al., (2020) [39]	Case report	N=1<br>M	75	Japan	Hyperintensity in the splenium of corpus callosum, suggesting mild encephalitis/encephalopathy with a reversible splenial lesion	Mild Alzheimer's disease
Helms et al., (2020) [74]	Observational study	N=58<br>(sex N.A.)	63	France	Neurologic signs:<br>-Agitation (N=40)<br>-Confusion (N=26)<br>-Corticospinal tract signs (N=39)<br>-Dysexecutive syndrome (N=14)<br>Brain MRI:<br>-Perfusion abnormalities (N=11)<br>-Leptomeningeal enhancement (N=8)<br>-Cerebral ischemic stroke (N=3)	Neurologic disorders (N=7): transient ischemic attack, partial epilepsy, mild cognitive impairment
Hjelmeseth & Skaare (2020) [40]	Case report	N=2<br>M=1<br>F=1	60	Norway	-F: anosmia<br>-M: ageusia<br>(only symptoms)	M: Billroth II surgery for gastric ulcers and atrial fibrillation

Hopkins et al., (2020) [75]	Observational cohort study	N=382<br>M=97<br>F=285	40–49	UK	-330 had complete anosmia<br>-44 had severe loss of smell	N.A.
Hornuss et al., (2020) [83]	Case-control study	N=45<br>F=20<br>M=25<br>CG=45	56±16.9<br><br><br>54±18.3	Germany	-Anosmia (N=18)<br>-Hyposmia (N=20)<br>-Normosmia (N=7)	N.A
Kandemirli et al., (2020) [65]	Retrospective study	N=235<br>M=21<br>F=6<br>(sex referred to patients who underwent MRI)	63 (34-87)	Turkey	50 patients developed neurologic symptoms, 27 of whom did MRI: in 12 patients MRI showed abnormalities:<br>-10 had cortical FLAIR signal abnormality<br>-2 had acute transverse sinus thrombosis and acute infarction in right MCA territory	12 with abnormal MRI: hypertension (6), coronary artery disease (1), atrial fibrillation (1), Addison’s disease (1), diabetes mellitus (5), chronic kidney disease (1)
Kadono et al., (2020) [41]	Case report	N=1<br>M	44	Japan	Seizures and brain edema	Cerebral venous thrombosis with acute hemorrhagic infarction and epilepsy
Karadaş et al., (2020) [78]	Prospective study	N=239<br>M=133<br>F=106	46.46±15.41	Turkey	83 patients displayed neurologic symptoms:<br>-headache (N=64)<br>-impaired consciousness (N=23)<br>-dizziness (N=16)<br>-cerebrovascular disorders (9)	N.A.

Kaya et al., (2020) [42]	Case report	N=1 M	38	Turkey	Bilateral occipital and frontal cortical white matter and splenium of corpus callosum FLAIR hyperintensities and DWI revealing vasogenic edema similar to PRES manifesting with cortical blindness, apathia and inability to respond to commands	None
Kishfy et al., (2020) [43]	Case report	N=2 M=1 F=1	58 M 67 F	USA	PRES	Patient 1: hyperlipidemia Patient 2: hypertension, obesity, type 2 diabetes mellitus
Lechien et al., (2020) [66]	Multicenter Retrospective study	N=417 M=154 F=263	36.9 ± 11.4	Belgium, France, Spain, Italy	-357 had olfactory dysfunctions -367 had gustatory dysfunctions	Respiratory insufficiency, neurological diseases, allergic rhinitis (more than 15%), asthma (almost 8%), high blood pressure (more than 6%), hypothyroidism, diabetes, CRS, renal failure, hepatic insufficiency, GERD, heart problems, depression, autoimmune diseases
Lee et al., (2020) [79]	Prospective study	N=3191 M=1161 F=2030	44.0 (25.0-58.0)	Korea	-Acute anosmia or ageusia was detected in 15.3% patients in the early phase of COVID-19 and in 15.7% asymptomatic/paucisymptomatic patients -STDs were more common among females and younger individuals	Hypertension, diabetes mellitus, cancer, end-stage renal failure, chronic lung diseases, congestive heart failure, cardiac diseases without congestive heart failure

					-Most patients with STDs recovered within 3 weeks	
Lu et al., (2020) [67]	Retrospective study	N=304<br>M=182<br>F=122	44 (33-59.25)	China	encephalopathy (N=8); seizure-like events (N=2); systemic or direct brain insults (N=84) (probably due to hypoxia N=77)	Acute cerebrovascular disease, TBI, CNS infection, hypoxia, shock, sepsis, multiple organ dysfunction,<br>hyperglycemia,<br>hypoglycemia,<br>hyponatremia,<br>hypocalcemia,<br>hypomagnesemia
Manganelli et al., (2020) [44]	Case report	N=3<br>M=1<br>F=2	60	Italy	-Patient 1 and 2: severe brainstem lesion<br>-Patient 3: punctiform gliotic foci in right pons	N.A.
Mao et al., (2020) [68]	Retrospective study	N=214<br>M=87<br>F=127	52.7±15.15	China	-Neurologic manifestations (N=78): dizziness (N=36), headache (N=28), impaired consciousness (N=16), acute cerebrovascular disease (N=6), ataxia (N=1), seizure (N=1)<br>-Patients with severe infection were older and with pre-existing pathologies, especially hypertension	Hypertension (51), diabetes (N=30), cardiac/cerebrovascular disease (N=15), malignancy (N=13), chronic kidney disease (N=6)
Mawhinney et al., (2020) [45]	Case report	N=1<br>M	41	UK	Acute mania	Congenital nystagmus
Melley et al., (2020) [46]	Case report	N=1<br>F	59	USA	Loss of appetite, dysgeusia, anosmia	Hypertension, hyperlipidemia, asthma

Morassi et al., (2020) [69]	Retrospective study	N=6<br>M=5<br>F=1	69 (57-82)	Italy	4 patients had ischemic strokes (3 died and 1 subject remained neurologically impaired) and 2 patients had hemorrhagic strokes (death)	Vascular risk factors, except for one patient
Moriguchi et al.,<br>(2020) [16]	Case report	N=1<br>M	24	Japan	-DWI revealed hyperintensity of right lateral ventricle and FLAIR images showed hyperintense signal changes in the right mesial temporal lobe and hippocampus with slight hippocampal atrophy, manifesting with transient generalized seizures and consciousness disturbance<br>-CSF was found positive for SARS-CoV-2	N.A.
Muhammad et al., (2020) [47]	Case report	N=1<br>F	60	Germany	-Left frontal aneurysmal subarachnoid haemorrhage from a ruptured pericallosal artery with loss of consciousness<br>-CSF was negative for SARS-CoV-2	N.A.
Noro et al., (2020) [48]	Case report	N=1<br>F	35	Brasil	Intracranial hypertension with prominent subarachnoid space around optic nerves and superior compression of the hypophysis	None
Ollarves-Carrero (2020) [49]	Case report	N=1<br>F	40	Spain	Anosmia	N.A.
Ottaviano et al.,<br>(2020) [50]	Case report	N=6<br>(sex N.A.)	N.A.	Italy	-Hyposmia and hypogeusia<br>-Symptoms were still present in 1 subject after 15 days	N.A.
Oxley et al., (2020) [51]	Case report	N=5<br>M=4<br>F=1	40.4	USA	Large-vessel ischemic stroke	Stroke (N=1), diabetes (N=1), hyperlipidemia and hypertension (N=1)

Petrescu et al., (2020) [70]	Retrospective study	N=36<br>M=27<br>F=9	43-97	France	EEG abnormalities in 42.5% of patients:<br>-10% moderate<br>-20% severe<br>-12.5% critical	No comorbidities in 2 patients. The others had past medical history of hypertension, diabetes mellitus, cardiomyopathy, renal failure and dementia, mostly
Poyiadji et al., (2020) [17]	Case report	N=1<br>F	late fifties	USA	MRI showed lesions within the bilateral thalami, medial temporal lobes and subinsular regions manifesting with altered mental status: the patient was diagnosed with acute necrotizing encephalopathy	N.A.
Radmanesh et al., (2020) [71]	Retrospective study	N=11<br>M=9<br>F= 2	53 (38-64)	USA	4 patients exhibited diffuse leukoencephalopathy, 1 exhibited microhemorrhages and 6 patients exhibited both	Hypertension, hyperlipidemia, type II diabetes, coronary artery disease, atrial fibrillation, gastrointestinal, deep vein thrombosis, cocaine use
Reichard et al., (2020) [52]	Case report	N=1<br>M	71	USA	Acute disseminated encephalomyelitis and neocortical microinfarcts	Ischemic heart disease due to coronary artery atherosclerosis
Rogg et al., (2020) [53]	Case report	N=1<br>M	59	USA	PRES	None
Sharifi-Razavi et al., (2020) [54]	Case report	N=1<br>M	79	Iran	Intracerebral hemorrhage in right hemisphere accompanied by intraventricular and subarachnoid haemorrhage manifesting with loss of consciousness and bilateral extensor plantar reflexes	None

Scullen et al., (2020) [72]	Retrospective study	N=76<br>M=40<br>F=36	59.8 (35-91)	USA	27 patients had neurologic features:<br>-encephalopathy (N=20)<br>-vasculopathy (N=5)<br>-acute necrotizing encephalopathy (N=2)	Hypertension (N=48), diabetes mellitus type 2 (N=39), obesity (N=20) and chronic kidney disease (N=17)
Shoskes et al., (2020) [55]	Case report	N=1<br>M	69	USA	Microhaemorrhages in corpus callosum, basal ganglia and brainstem	Hypertension, chronic kidney disease and hypothyroidism
Somani et al., (2020) [56]	Case report	N=2<br>F	49 F<br>73 F	USA	De novo status epilepticus	-Patient 1: rheumatoid arthritis, schizoaffective and conversion disorders<br>-Patient 2: hypertension, diabetes mellitus, chronic kidney disease and skull base encephalocele
Speth et al., (2020) [80]	Prospective, cross-sectional study	N=103<br>M= 50<br>F=53	48.5±15.9	USA	-63 patients displayed STDs<br>-severity of anosmia correlated with severity of dysgeusia<br>-STDs negatively correlated correlated with older age and positively correlated with female sex	Allergic rhinitis or hay fever (N=36),<br>chronic rhinosinusitis or polyps (N=1),<br>asthma (N=13)
Spinato et al., (2020) [73]	Retrospective study	N=202<br>M=97<br>F=105	56 (20-89)	Italy	-STDs (N=130)<br>-More frequent in women	None (N=89)

Tunç et al., (2020) [57]	Case report	N=4<br>M=2<br>F=2	45-77	Turkey	Acute ischemic stroke	Diabetes (N=1), hypertension (N=3)
Vaira et al., (2020a) [76]	Observational study	N=72<br>M=27<br>F=45	49.2±13.7	Italy	53 patients reported STDs:<br>-30 patients had both anosmia and dysgeusia	N.A.
Vaira et al., (2020b) [77]	Multicenter cohort study	N=345<br>M=146<br>F=199	48.5±12.8	Italy	256 patients reported STDs:<br>-203 reported both anosmia and dysgeusia<br>-22 had isolated anosmia<br>-30 had isolated dysgeusia	N.A.
Viguier et al., (2020) [58]	Case report	N=1<br>M	73	France	Acute ischemic stroke complicating common carotid artery thrombosis manifesting with aphasia and right hemiparesis	None
Wong et al., (2020) [59]	Case report	N=1<br>M	40	UK	Inflammatory rhombencephalitis/myelitis manifesting with diplopia, oscillopsia, limb ataxia, altered sensation in right arm, hiccups when eating or drinking, unsteady gait, nystagmus	Closed angle glaucoma and hypertension
Ye et al., (2020) [60]	Case report	N=1<br>M	N.A.	China	-Encephalitis<br>-CSF was negative for SARS-CoV-2	N.A.
Zanin et al., (2020) [61]	Case report	N=1<br>F	54	Italy	-Anosmia and ageusia<br>-Focal sensorimotor deficits<br>-Two seizures starting from right frontotemporal region and diffusing in homologous contralateral hemisphere	ACA aneurysm

					-Demyelinating lesions in periventricular white matter, bulbo-medullary junction and in both the cervical and dorsal spinal cord<br><br>-CSF was negative for SARS-CoV-2	
Zhai et al., (2020) [62]	Case report	N=1<br><br>M	79	China	Ischemic stroke	None
Zoghi et al., (2020) [63]	Case report	N=1 M	21	Iran	Encephalomyelitis	N.A.

Notes: ACA, anterior communicating artery; CG, control group; CNS, central nervous system; COPD, chronic obstructive pulmonary disease; COVID-19, coronavirus disease-19; CRS, chronic rhinosinusitis; CSF, cerebrospinal fluid; CT, computerized tomography; DWI, diffusion weighted imaging; EEG, electroencephalography; F, female; FLAIR, fluid-attenuated inversion recovery; GERD, gastroesophageal reflux disease; H&H, Hunt and Hess; M, male; MCA, middle cerebral artery; MRI, magnetic resonance imaging; N.A., not applicable; PRES, posterior reversible leukoencephalopathy syndrome; SAH, subarachnoid haemorrhage; SARS-CoV-2, Severe Acute Respiratory Syndrome-Coronavirus-2; STDs, smell and taste disorders; TBI, traumatic brain injury.

Table 2. Studies concerning COVID-19 effects on the Peripheral Nervous System.

Article	Study design	Sample	Age	Country	Neurological Complications	Prior Medical Conditions
Abdelnour et al., (2020) [86]	Case report	N=1<br><br>M	69	UK	Numbness on both legs, reduced muscular power, gait ataxia	Hypertension, type 2 diabetes mellitus, mild COPD
Alberti et al., (2020) [87]	Case report	N=1<br><br>M	71	Italy	-Guillain-Barré syndrome<br><br>-CSF was found negative for SARS-CoV-2	Hypertension, abdominal aortic aneurysm, lung cancer
Coen et al., (2020) [88]	Case report	N=1 M	70	Switzerland	Acute inflammatory demyelinating polyneuropathy, the most common subtype of Guillain-Barré syndrome	None

Dinkin et al., (2020) [89]	Case report	N=2<br>M<br>F	36 M<br>71 F	USA	-Miller Fisher syndrome and ophthalmoparesis from cranial nerve palsy<br>-CSF was found negative for SARS-CoV-2	M: infantile strabismus<br>F: hypertension
Farzi et al.,<br>(2020) [90]	Case report	N=1<br>M	41	Iran	Guillain-Barré syndrome	Type II diabetes mellitus
Gutiérrez-Ortiz et al., (2020) [91]	Case report	N=2<br>M<br>F	50 M<br>39 F	Spain	-Miller Fisher syndrome and polyneuritis cranialis<br>-CSF was found negative for SARS-CoV-2	M: bronchial asthma<br>F: none
Homma et al., (2020) [92]	Case report	N=1<br>F	35	Japan	Facial nerve palsy and olfactory disturbance	None
Hutchins et al., (2020) [93]	Case report	N=1<br>M	21	USA	Guillain-Barré syndrome	Hypertension, diabetes and class I obesity
Karadaş et al., (2020) [78]	Prospective study	N=239<br>M=133<br>F=106	46.46±15.41	Turkey	Trigeminal neuralgia (N=8), glossopharyngeal neuralgia (N=9), vagoglossopharyngeal neuralgia (N=2), muscle pain (N=36), restless leg syndrome (N=4) and Guillain-Barré syndrome (N=1)	N.A.
Lantos et al., (2020) [94]	Case report	N=1<br>M	36	USA	Miller Fisher syndrome	Left eye strabismus
Mao et al., (2020) [68]	Retrospective study	N=214<br>M=87<br>F=127	52.7±15.5	China	Neurologic symptoms (N=78): taste (N=12), smell (N=11); vision impairment (N=3); nerve pain (N=5); skeletal muscle pain (N=23)	Hypertension (N=51), diabetes (N=30), cardiac or cerebrovascular disease (N=15), cancer (N=13),

						chronic kidney disease (N=6)
Ottaviani et al., (2020) [95]	Case report	N=1<br>F	66	Italy	-Guillain-Barré syndrome<br>-CSF was found negative for SARS-CoV-2	N.A.
Padroni et al., (2020) [96]	Case report	N=1<br>F	70	Italy	Guillain-Barré syndrome	N.A.
Rana et al., (2020) [97]	Case report	N=1<br>M	54	USA	Guillain-Barré syndrome	Hypertension, hyperlipidemia, restless leg syndrome, chronic back pain
Sedaghat & Karimi, (2020) [98]	Case report	N=1<br>M	65	Iran	Guillain-Barré syndrome	N.A.
Scheidl et al., (2020) [99]	Case report	N=1<br>F	54	Germany	Guillain-Barré syndrome	None
Toscano et al., (2020) [100]	Case report	N=5<br>M=4<br>F=1	58.4	Italy	-Guillain-Barré syndrome<br>-CSF was found negative for SARS-CoV-2	N.A.
Virani et al., (2020) [101]	Case report	N=1<br>M	54	USA	Guillain-Barré syndrome	N.A.
Webb et al., (2020) [102]	Case report	N=1<br>M	57	UK	Guillain-Barré syndrome	Untreated hypertension and psoriasis
Wei et al., (2020) [103]	Case report	N=1<br>M	62	China	Oculomotor nerve palsy	Alcohol and tobacco use, type II diabetes mellitus, hypertension, lacunar infarction
Zhao et al., (2020) [104]	Case report	N=1<br>F	61	China	-Guillain-Barré syndrome<br>-CSF was found negative for SARS-CoV-2	N.A.

Notes: COPD, chronic obstructive pulmonary disease; CSF, cerebrospinal fluid; F, female; M, male; N.A., not applicable; SARS-CoV-2, Severe Acute Respiratory Syndrome-Coronavirus-2.
